# Integrated clinical and metabolomic analysis identifies molecular signatures, biomarkers, and therapeutic targets in primary angle closure glaucoma

**DOI:** 10.3389/fmolb.2024.1421030

**Published:** 2024-08-09

**Authors:** Vishnu Kannan, Sai Krishna Srimadh Bhagavatham, Rajesh Babu Dandamudi, Haripriya Kunchala, Sivateja Challa, Abdulrahman I. Almansour, Ashish Pargaonkar, Sujith Kumar Pulukool, Anuj Sharma, Venketesh Sivaramakrishnan

**Affiliations:** ^1^ Disease Biology Lab, Department of Biosciences, Sri Sathya Sai Institute of Higher Learning, Prasanthi Nilayam, Andhra Pradesh, India; ^2^ Department of Chemistry, Sri Sathya Sai Institute of Higher Learning, Prasanthi Nilayam, Andhra Pradesh, India; ^3^ Department of Ophthalmology, Sri Sathya Sai Institute of Higher Medical Sciences, Prasanthi Gram, Andhra Pradesh, India; ^4^ Department of Chemistry, College of Science, King Saud University, Riyadh, Saudi Arabia; ^5^ Application Division, Agilent Technologies Ltd., Bengaluru, India

**Keywords:** glaucoma, metabolomics, inflammation, PACG, immuno-metabolism

## Abstract

**Background:**

Glaucoma is the leading cause of permanent blindness. Primary angle closure glaucoma (PACG) is diagnosed only after the onset of symptoms and can result in irreversible blindness despite the standard intraocular pressure (IOP) reduction therapy. The identification of potential biomarkers associated with prognosis will help improve disease management. This study aimed to identify mechanisms associated with disease progression, potential biomarkers, and therapeutic targets of PACG.

**Methods:**

The clinical data assessment of IOP, cup/disc ratio (CDR), Retinal Nerve Fiber Layer (RNFL) thickness of control, and PACG group were collected and analyzed for significant differences. The ATP levels were estimated, and targeted metabolomic analysis was performed on aqueous humor and cytokines in plasma. The pathways obtained from the metabolomics data set were compared with those obtained for data sets from the literature. Clinical parameters were correlated with cytokine levels. Targeted metabolomic analysis of cell culture supernatant from TNFα-treated N9 microglia was carried out, and overlap analysis was performed with data obtained from PACG patients.

**Results:**

Elevated IOP, CDR, ATP, cytokines, and reduced RNFL thickness were found in PACG compared to controls. Analysis of PACG and TNFα-treated N9 microglial cell culture supernatant shows activation of immuno-metabolites. The metabolic pathways of PACG, TNFα, and ATP-treated microglia from the literature show considerable overlap. Biomarker analysis identified clinical parameters, ATP, cytokines, and immuno-metabolites.

**Conclusion:**

This study shows an association between elevated levels of ATP, cytokines, immuno-metabolism, and potential microglial inflammation with disease progression, rendering these levels potential biomarkers. P2 receptors, cytokines, and IDO1/2 could be potential therapeutic targets.

## 1 Introduction

Glaucoma is estimated to affect approximately 111 million people by 2040 and is a leading cause of permanent blindness (Tham et al., 2014). In India, an estimated 12 million people are affected by glaucoma (Thomas, 2011). Though primary angle closure glaucoma (PACG) is less prevalent, it is difficult to manage (Krishnadas, 2019). Elevated IOP and cup/disc ratio, reduced retinal nerve fiber layer (RNFL) thickness, and gradual peripheral vision loss are associated with PACG (Ahmad, 2018). Though the standard of care treatment for PACG is to employ either medication or surgery for reducing elevated IOP, the disease progresses with a deterioration in visual function (Sheybani et al., 2020). Hence, mechanisms leading to axonal injury resulting in the death of retinal ganglion cells (RGC) need to be elucidated to identify potential biomarkers, therapeutic targets, and therapeutic agents for better disease management.

Earlier studies have shown a positive correlation between elevated IOP and ATP levels in the aqueous humor (Zhang et al., 2007). Elevated levels of ATP induce inflammation, leading to the secretion of cytokines and retinal ganglion cell death (Niyadurupola et al., 2013). Both microglia and RGC constitutively express P2 receptors, and inhibition of ATP receptors is found to be neuroprotective (Niyadurupola et al., 2013; Sugiyama, 2014). Few isolated studies have shown that cytokines like TNFα or TGFβ are elevated in PACG (Tong et al., 2017). Anti-TNFα antibodies have exhibited therapeutic potential, although they do have side effects (Clark and Vissel, 2016).

Previous investigations have mainly focused on PACG-associated risk factors like genetic and metabolic factors in different populations. Studies have demonstrated that single nucleotide polymorphisms (SNPs) in many genes are associated with PACG (Amerasinghe et al., 2011). Metabolomic analysis of PACG serum samples showed significant changes in the levels of free fatty acids like palmitoleic acid, linoleic acid, and arachidonic acid as important metabolites (Rong et al., 2017). Considering the inflammatory nature of the disease, potentially elevated levels of immuno-metabolites may be associated with PACG.

Microglia, immune cells found in both the brain and retina, play a role in glaucoma, and their activation is associated with the disease (Rong et al., 2017). *In vitro* studies using microglial primary culture or cell lines like BV2 and N9, similar to primary culture, have delineated the underlying mechanisms associated with glaucoma (Pulukool et al., 2021). Microglial activation by cytokine and inflammatory agents like LPS or ATP are shown to invoke inflammation, metabolic remodeling, and inflammatory response (Kannan et al., 2013; Pulukool et al., 2021).

The current study deals with an integrative analysis of clinical parameters, plasma cytokine profiling, ATP measurements, and targeted metabolomic analysis of PACG patient aqueous humor using age- and gender-matched controls to determine the possible association between inflammation and metabolic remodeling with the disease. The pathways obtained from our metabolic study were further compared with a rat model of PACG and showed considerable concordance. Furthermore, the *in vitro* N9 microglial cell culture model is employed to show that TNFα-induced metabolic remodeling involving immuno-metabolism is similar to that observed in PACG. Because our previous studies demonstrated that TNFα-induced inflammation is dependent on ATP signaling, we compared the pathways obtained in PACG patients with TNFα- or ATP-treated N9 microglial cells and found significant overlap. Taken together, our results on microglia show activation of immuno-metabolism and its association with disease.

Overall, our results show the sequelae of events/associations like changes in various clinical parameters like IOP, CDR, a reduction in RNFL thickness, ATP, cytokine levels, and metabolic remodeling involving immuno-metabolites with PACG. Cell culture studies using N9 microglia cells reiterate an involvement of TNFα in inducing metabolic remodeling involving immuno-metabolites with implications for the progression of the disease.

## 2 Materials and methods

**FIGURE 1:**
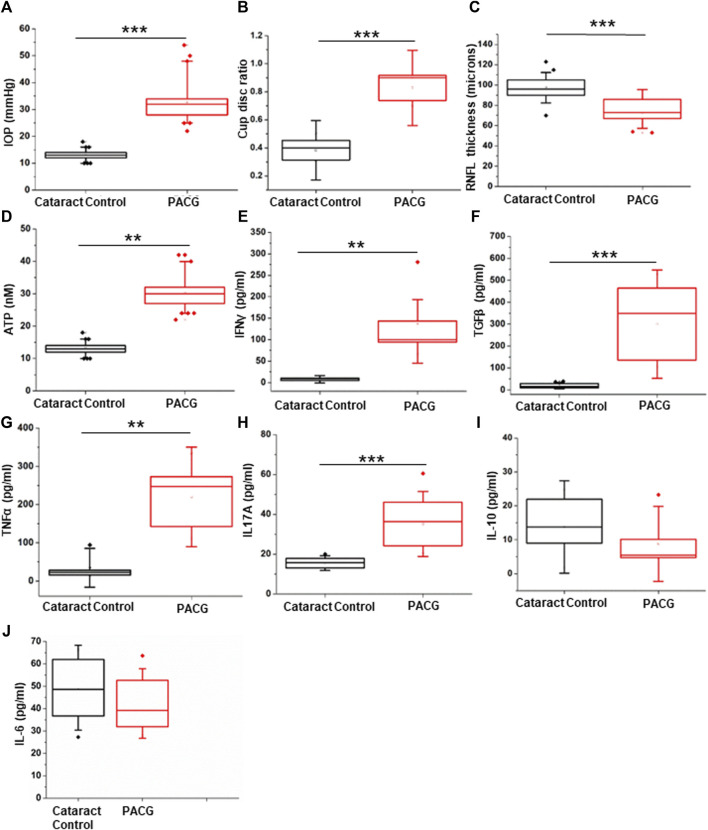
Assessment of clinical parameters: **(A)** IOP measurement in the PACG cohort compared to the control. **(B)** Cup-disc ratio in the PACG group compared to the control. **(C)** RNFL thickness in the PACG cohort compared to the control eyes. **(D)** ATP levels in the aqueous humor of control and PACG cohort. Cytokine levels of **(E)** IFNγ, **(F)** TGFβ, **(G)** TNFα, **(H)** IL17A, **(I)** IL-10, and **(J)** IL-6 in the blood plasma of the control group and the PACG cohort. Statistical analysis (p-values) was calculated using the Mann–Whitney U test. * for p < 0.05, ** for p < 0.01, and *** for p < 0.001. For n numbers and the results provided as mean ± s.d., refer to the text.

The studies were conducted in the Department of Ophthalmology at Sri Sathya Sai Institute of Higher Medical Sciences, Prasanthigram, India, following the collection of informed consent. The prospective patient group consisted of cataract controls (n = 7) and PACG patients (n = 9), while the retrospective data of cataract controls (n = 25) and PACG patients (n = 20) were obtained for the period 2014–2019 from the hospital information system, all age and gender-matched. The combined clinical data of prospective and retrospective cohorts are provided in Figure 1 and separate analyses of prospective as retrospective data are provided in Supplementary Figures S1, S2 as outlined in the results section. The approval from the Institutional Ethics Committee (“Sri Sathya Sai Institute of Higher Learning, Institutional Ethics Committee with the Registration No: ECR/616/Inst/AP/2014/RR-17”) was obtained, and the tenets of the 1964 Declaration of Helsinki was strictly adhered to. The patients were recruited following the exclusion and inclusion criteria mentioned below.

### 2.1 Inclusion and exclusion criteria

Inclusion: PACG patients with an IOP of >21 mm Hg measured in duplicate and findings in the optic disc that are typical of glaucoma. Patients with an IOP >21 mm Hg in the affected eye and who were diagnosed with other forms of glaucoma, as well as those with conditions like surgery or trauma, inflammatory eye diseases, systemic inflammatory disease, and diseases of the ocular surface, were excluded. The control group included cataract patients without glaucoma who underwent cataract surgery.

### 2.2 Procedures followed in the study

The control and the patients recruited for the study underwent a thorough examination to obtain the complete medical history. The various parameters, such as best-corrected visual acuity for far and nearsightedness, were performed using Snellen’s denomination, while a slit lamp (BQ900 HAGG-STREIT International) was employed for evaluating the features of the anterior segment. The IOP measurement was performed using a Goldmann applanation tonometer (AT 900). Gonioscopy and OCT (Spectral OCT SLO; OPKO Health Inc., United States) were also performed. The tests and analysis were performed as described previously (Pulukool et al., 2021). Correlation analysis was performed on both prospective and retrospective data sets obtained for IOP, CDR, and RNFL thickness. Planned cataract surgery was performed on all the patients, including PACG patients. Samples of serum and plasma (5 mL each; fasting blood) were collected before surgery for analysis. Standard of care treatment was followed for sample collection and treatment. Parenthesis during the surgery was performed using a 26 G needle on a tuberculin syringe following standard procedures. The aqueous humor from the patients was obtained and stored at −80°C after flash freezing until further analysis, as described previously (Pulukool et al., 2021).

### 2.3 ATP measurements

The ATP concentration in aqueous humor samples was measured using the luciferin-luciferase reaction (ThermoFisher Scientific Cat. No. A22066) as described previously (Pulukool et al., 2021) with a Luminometer (Berthold). In brief, 90 µl of standard reaction mixture was mixed with 10 µl of double distilled water to measure background luminescence. For the actual measurement, 90 µL of the standard reaction mixture was mixed with 10 µL of diluted ATP standard solution or aqueous humor sample to initiate the reaction. ATP standards were prepared according to the manufacturer’s instructions. The concentration of ATP was measured using the manufacturer’s instructions. Only prospective data were used to correlate IOP with ATP.

### 2.4 Enzyme-linked immunosorbent assay (ELISA)

Plasma samples from the cataract controls and the PACG group were used for cytokine profiling. The levels of cytokines TGFβ (Cat. No. CHC 1683), TNFα (Cat. No. CHC 1753), IFNγ (Cat. No. CHC 1233), and IL-10 (Cat No. 1323) were determined using commercially available ELISA kits (Thermo Fisher Scientific) while IL-17A (Cat. No. 900-M84) and IL-6 (Cat. No. 900-M16) were estimated using commercially available ELISA kits (Pepro Tech). Antibody dilutions were performed as per the manufacturer’s instructions. In brief, the 96-well microtiter plates were coated with capture antibody (Nunc MaxiSorp Flat bottom, Cat No. 442404) and further processed by following the steps outlined in the manufacturer’s instructions. The ELISA was performed as outlined in the manufacturer’s instructions. The calculations were performed on the data using a log-log or a 4-parameter for curve fitting to obtain the results.

### 2.5 Cell culture, chemicals, and reagents

Cell culture experiments were performed using the Murine N9 microglia cells. N9 microglia cells were kindly provided by Anirban Basu from the National Brain Research Centre in Haryana (Mukherjee et al., 2018). Cell culture of N9 microglia was carried out using the RPMI media (Gibco) supplemented with 10% heat-inactivated South American origin fetal bovine calf serum from Invitrogen with 100ug/mL each of streptomycin and penicillin in a humidified atmosphere with 5% CO_2_ at 37°C. The cells for the experiments were used within eight passages. The culture plates and T25 flasks were procured from Corning and Nunc, respectively. Himedia trypsin was procured, and all other chemicals, unless otherwise specified, were procured from Merck.

### 2.6 Metabolomic analysis

The procedure employed for metabolomic analysis is as described previously (Pulukool et al., 2021). In brief, 2.5 µL of labeled internal standards (L-tryptophan 15N_2_, zeatine, L-arginine, L-jasmonic acid) was spiked to 50 µL of aqueous humor, which was further diluted with 200 µl of distilled water. To 50 µL of cell culture supernatant, 150 µL of internal standards were prepared, and 50% methanol was added and incubated in ice for 30 min. The samples were then sonicated for 15 min and centrifuged at 10,000 rpm for 2 min at 4°C.

The processed aqueous humor sample was filtered using a 3 kDa cutoff Amicon filter (Merck Millipore Cat. No. UFC500396), and the filtrate was dried using a SpeedVac. The resuspension of the residue was done in 100 µL of 0.1% formic acid (Sigma). The resuspended extract was vortexed and centrifuged at 13,000 rpm for 5 min, and 2 μL of the prepared extract was injected in an Agilent 6490 triple quadrupole mass spectrometer. Similarly, the processed cell culture supernatant samples were passed through a 3 kDa Amicon filter and processed as described previously. A 5 µl volume of the filtrate was injected in an Agilent 6490 triple quadrupole mass spectrometer. For the identified metabolites, the area under the peak was used for quantification. The quantified peak values obtained for metabolites were further normalized with those obtained for the internal standard (L-jasmonic acid).

An X-Bridge amide column (part no. 186004868, Waters, United States) in the negative ionization mode was used for sample analysis. The mobile phase is constituted by solvent A, which is 20 mM ammonium acetate (Optima LC/MS, Cat. No. A11450 Fisher Scientific, United States) in a 95:5 water:acetonitrile system, pH 9.0, while solvent B is 100% acetonitrile (ACN). A gradient of solvent A and Solvent B was employed at a constant flow rate of 0.3 mL/min. The details of the gradient used are provided in Supplementary Table S1. The results were analyzed using MetaboAnalyst. Non-parametric statistical analysis was performed with a false discovery rate (FDR) cutoff value of 0.25 to obtain the significant differential metabolites for PACG to cataract controls. Random forest, pathways, and biomarker analyses were also carried out using MetaboAnalyst.

### 2.7 Comparative analysis with the published literature

The pathways obtained from a metabolomic analysis of our PACG patient cohort were compared with the metabolomic data sets of a hyaluronate-induced rat disease model (Mayordomo-Febrer et al., 2015). MetaboAnalyst 6.0 was used for the pathway analysis to obtain the list of deregulated pathways (Pang et al., 2024). The PACG proteomic data were collected from the literature (Liu et al., 2021; Semba et al., 2021) using Enrichr (Chen et al., 2013; Kuleshov et al., 2016), and the differential genes were grouped into pathways. After proteomic analysis, the pathways obtained from individual datasets were pooled and then compared with the pathways obtained from metabolomic datasets. The significant pathways obtained for our PACG metabolomics were overlapped with the pathways obtained for metabolomic data sets of rat model of disease and PACG proteomic data from literature using the Venn Diagram tool (https://bioinfogp.cnb.csic.es/tools/venny/index.html).

### 2.8 Statistical analysis

The clinical parameters, ATP and cytokine levels were analyzed for statistical significance with the Mann–Whitney U test. The results are provided as box plots (mean ± SD). Pearson’s correlation was performed between two parameters, and the correlation coefficient was classified following standard procedures. Metabolomic data analysis was carried out using an online tool, MetaboAnalyst 5.0 (Pang et al., 2021), and internal standards were used for normalization. The Mann–Whitney test coupled with false discovery rate correction using MetaboAnalyst was used to determine the significant metabolites. The clustering of samples was performed using principal component analysis (PCA) and partial least-squares discriminant a*nalysis* (PLS-DA) analysis. Random forest analysis was performed on metabolomics data sets to stratify PACG patients from controls based on the mean decrease in accuracy. Biomarker analysis was performed using the receiver operating characteristic analysis (ROC) analysis provided in MetaboAnalyst. The true positive and negative rates representing sensitivity and specificity, respectively, were analyzed at a threshold of 0.25, and the metabolites were arranged based on the values obtained for the area under the curve (AUC) at 95% confidence.

## 3 Results

### 3.1 Clinical analysis showed increased levels of IOP and cup-disc ratio with reduced RNFL thickness in PACG patients

The diagnosis and recruitment of the PACG and cataract control patients for the study were performed following the procedures outlined in the methods. The results of all analyses are expressed as mean ± s.d. (standard deviation).

The RNFL thickness (Control: 97.866 ± 13.373 µm, n = 11; PACG: 72.363 ± 13.116 µm, n = 11) was determined using an OCT, and the cup-disc ratio (control: 0.3833 ± .0707, n = 18; PACG: 0.827 ± 0.089, n = 18) was determined using slit lamp. Tonometry was used to determine IOP (control: 12.5 ± 1.723 mmHg, n = 15; PACG: 35.44 ± 8.610 mmHg, n = 15) on both eyes. Significantly elevated levels of IOP (p = 0.00001) and CDR (p = 0.00001) were found in PACG patients compared to cataract controls (Figures 1A,B). Significantly reduced RNFL thickness (p = 0.00116) was observed in PACG patients compared to cataract controls (Figure 1C). Separate graphs of prospective and retrospective clinical data sets, along with the sample size (n number) in the legend, are provided as Supplementary Figure S1 (prospective) and Supplementary Figure S2 (retrospective). The IOP, CDR, and RNFL thickness parameter values for the eyes in the Control group were within the normal range. The cup/disc ratio and the IOP displayed a strong positive Pearson’s correlation of 0.728 (control n = 32 and PACG n = 28), while the IOP and RNFL thickness displayed a strong negative correlation of −0.535 (control n = 17 and PACG n = 18). The results from our patient cohort representing the Indian population comply with the parameters that were reported for PACG previously (Zhu et al., 2019).

### 3.2 The PACG patients exhibited elevated levels of ATP in the aqueous humor

Earlier studies show that increased levels of IOP exert mechanical stress, which is correlated with elevated ATP concentration in the aqueous humor (Pulukool et al., 2021). The results of ATP measurements are expressed as mean ± s.d. The aqueous humor ATP concentrations (p = 0.0043) were significantly higher in PACG patients (n = 9, 25.745 ± 9.756 nM) than in controls (n = 7, 5.865 ± 3.756 nM) (Figure 1D). Pearson’s correlation of IOP and ATP levels showed a significantly strong positive value of 0.7856. Because elevated extracellular ATP is known to invoke an inflammatory response, cytokine measurements were performed in the patients’ plasma.

### 3.3 Levels of inflammatory cytokine are elevated in the plasma of PACG patients

Cytokine level measurements were performed in seven controls and nine PACG plasma samples and expressed as mean ± s.d. The cytokine ELISA of IFNγ (control: 8.128 ± 2.982 pg/ml; PACG: 136.886 ± 85.439 pg/ml), TGFβ (control: 20.092 ± 9.958 pg/ml; PACG-300.27 ± 164.69 pg/ml), TNFα (control: 34.942 ± 33.733 pg/ml; PACG: 220.162 ± 86.821 pg/ml), IL-17A (control: 15.49 ± 2.416 pg/ml; PACG: 35.146 ± 10.884 pg/ml), and IL-10 (not different from control) and IL-6 (not different from control) were carried out on the plasma of PACG patients and controls as enunciated in methods. Significantly elevated levels of IFNγ (p = 0.0027), TGFβ (p = 0.00076), TNFα (p = 0.00438), and IL17A (p = 0.00026) were observed in PACG compared to controls (Figures 1E–H). Changes in the levels of IL-10 and IL-6 were found to be insignificant (Figures 1I,J). The cytokine profile showed an inflammatory bias, consistent with the association of inflammation and significantly elevated levels of TNFα and IFNγ with PACG.

### 3.4 The aqueous humor metabolic profile shows deregulated metabolic pathways with potential implications for PACG

**FIGURE 2:**
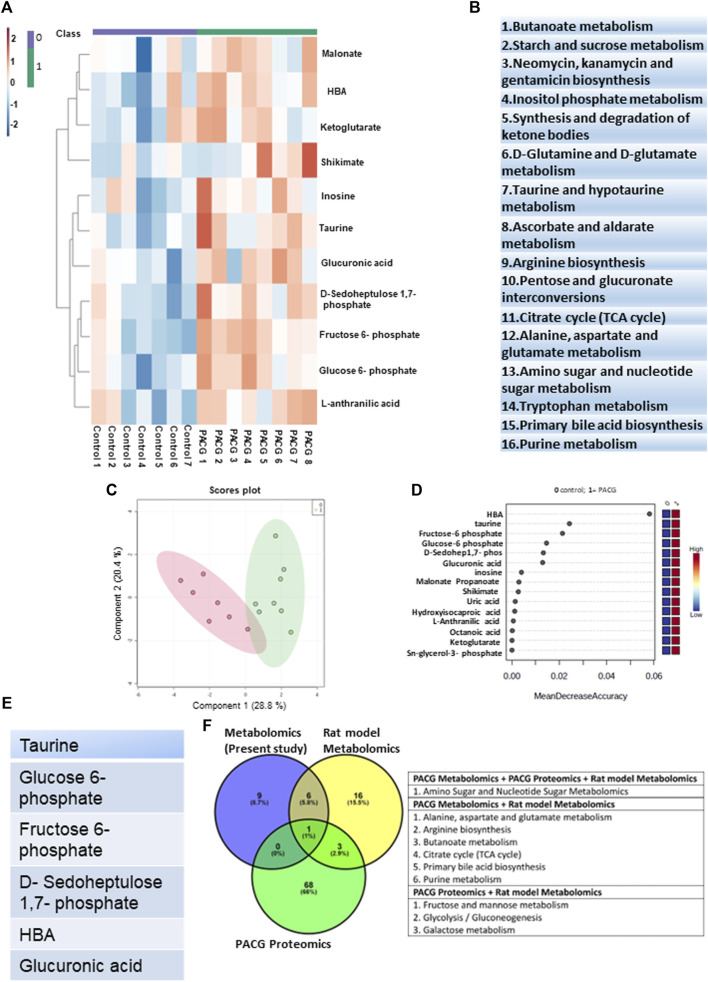
Targeted metabolomic analysis of aqueous humor in PACG compared to controls: **(A)** Unsupervised heat map of metabolomic analysis of control and PACG patient cohorts. Eleven significant differential metabolites were obtained at an FDR of 0.25. **(B)** List of pathways showing significant differential metabolites belonging to PACG binned at an FDR of 0.25. **(C)** Score plot showing an unbiased PLS-DA clustered the samples into two different groups. **(D)** Random forest analysis distinguishing PACG from controls. **(E)** Ranking of significant metabolites based on biomarker analysis with area under curve (AUC) values above 0.8. **(F)** Commonality analysis to identify pathways obtained from our own metabolomics analysis, as well as those obtained from rat model metabolomics and PACG pooled proteomic datasets from the literature. Panels 2A, C, and D were generated using MetaboAnalyst 5.0 (Version 5.0; URL: https://www.metaboanalyst.ca/).

Targeted metabolomic analysis was performed on aqueous humor from PACG (n = 8) and cataract controls (n = 7) using multiple reaction monitoring (MRM) in the negative mode. The relative levels of 76 metabolites in the clinical samples were determined, and 23 were detected. The results were analyzed using the online tool MetaboAnalyst. Non-parametric analysis of significant differential metabolites identified 11 metabolites at an FDR cutoff of 0.25 between PACG and cataract controls; these results are provided as a heat map (Figure 2A). The significant differential metabolites obtained include glucose-6-phosphate, fructose-6-phosphate, D-sedoheptulose 1,7-phosphate, etc. (Supplementary Figure S3). These significant metabolites were grouped into 16 pathways (Figure 2B), which include the butanoate metabolism, the starch and sucrose metabolism, the glutamine and glutamate metabolism, the pentose and glucuronate metabolism, the purine metabolism, the TCA cycle, the inositol phosphate metabolism, the tryptophan metabolism, etc. (Figure 2C). These deregulated pathways have a potential role in inflammation, cytokine secretion, etc (Huang et al., 2014). The PLS-DA analysis resulted in the samples being clustered into two different groups, which are represented using a score plot (Figure 2C). PCA analysis also resulted in the samples being clustered into two different groups, which are also represented using a score plot (Supplementary Figure S3). Our results showed that the PLS-DA cluster analysis displayed better separation of the two clustered samples. Furthermore, cross-validation was performed, which resulted in good model parameters (R2 = 0.7875, Q2 = 0.6348) (Supplementary Figure S3).

Random forest analysis of the 11 differential metabolites with 5,000 trees identified HBA, taurine, glucose-6-phosphate, and fructose-6-phosphate as the top metabolites that distinguish PACG from controls (Figure 2D). Biomarker analysis identified taurine, fructose 6-phosphate, D-sedoheptulose 1,7 phosphate, glucose-6-phosphate, HBA, and glucuronic acid (Figure 2E) as the biomarkers with high reliability with a value above 0.8 for AUC (Supplementary Figure S3). One of the PACG samples was an outlier and was removed from the analysis. The results, including the outlier PACG sample, are provided in Supplementary Figure S4.

### 3.5 Comparison of the pathways obtained from metabolomics analysis of our PACG patient cohort with metabolomics datasets of a rat model of disease and proteomic data showed overlapping pathways

We further analyzed the metabolomics data sets of hyaluronate-induced rat models of glaucoma, which are representative of PACG, from the literature (Mayordomo-Febrer et al., 2015). The histopathology of the hyaluronate-induced glaucoma showed a resemblance to PACG (Bouhenni et al., 2012). The analysis of metabolomics data sets using the significant differential metabolites from hyaluronic acid injected with respect to the saline-injected rat model showed 10 metabolites, including glucose, creatine/creatinine, citrate, acetoacetate, proline, glutamate/glutamine, lysine, and alanine. However, because glutamine was not significant, the glutamate/glutamine was taken as a glutamate peak (Mayordomo-Febrer et al., 2015). In addition, the data set also contained an unidentified peak for metabolite as well as a peak for fatty acids, which were not included. Analysis of pathways using these differential metabolites grouped them into 26 metabolic pathways. These pathways include the nitrogen metabolism, the metabolism of many amino acids, butanoate, biotin, fructose and mannose, pantothenate and CoA biosynthesis, and the porphyrin, nucleotide, and steroid metabolism (Supplementary Figure S5A).

We then analyzed the proteomic data set of PACG using significant differential proteins from Liu et al. (2021) and Semba et al. (2021). Our analysis using the significant differential proteins identified 72 significant pathways. These pathways include immune, signaling, and metabolic pathways, as well as pathways associated with neurodegenerative and infectious diseases (Supplementary Figure S5B).

Furthermore, we performed commonality analysis to identify pathways obtained from our own metabolomics analysis as well as those obtained from rat model metabolomics and PACG proteomic data sets from the literature. The commonality analysis showed amino sugar and nucleotide sugar pathways to be common among all three datasets (Figure 2F). The PACG proteomic and rat model metabolomics data sets showed three common pathways (Figure 2F). These pathways include the fructose and mannose metabolism, the galactose metabolism, and glycolysis/gluconeogenesis (Figure 2F). The pathways from the rat model metabolomics data set and our PACG metabolomics data sets showed six common pathways (Figure 2F). These pathways include the alanine, aspartate, and glutamate metabolism; arginine biosynthesis; the butanoate metabolism; the citrate cycle; primary bile acid biosynthesis; and the purine metabolism.

The results from these different data sets show that pathways associated with the disease among different taxa, study settings, and techniques used showed considerable overlap. These results demonstrate that despite a low cohort of PACG patients, our metabolomics data analysis could capture the tenets of metabolic remodeling associated with PACG.

### 3.6 Correlation analysis of clinical parameters, ATP, cytokines, and significant differential metabolites show strong correlations with potential implications for disease

Pearson’s correlation analysis of the RNFL shows a strong negative correlation with cytokines like TNFα, IFNγ, TGF, and IL17A, ATP levels, and CDR. The IOP strongly correlated with IL17A, CDR, ATP, and fructose-6-phosphate. The CDR strongly correlated with fructose-6-phosphate, TNFα, IFNγ, TGFβ, and IL17A. The TNFα showed a strong positive correlation with RNFL, IFNγ, TGFβ, IL17A, ATP, and CDR. The IFNγ and TGFβ strongly correlated with RNFL thickness, ATP, CDR, and the remaining cytokines. The IFNγ strongly correlated with RNFL thickness, fructose-6-phosphate, CDR, and the remaining cytokines. ATP levels strongly correlated with RNFL thickness, IOP, TNFα, TGFβ, IFNγ, and fructose-6-phosphate. Fructose-6-phosphate correlated with IOP, CDR, IL17A, ATP, LAC, D-sedoheptulose 1,7 phosphate, and glucose-6-phosphate. These results show that the clinical parameters correlate with cytokines, which, in turn, correlate with immuno-metabolites in PACG. The result of significant differential metabolites using Pearson’s correlation analysis is provided in Table 1. The complete table and the values are provided in Supplementary Table S2.

**TABLE 1:** Results of Pearson’s correlation analysis.

	RNFL	IOP	CDR	TNFα	IFNγ	TGFβ	IL-17A	ATP	LAC	F6P	DSED	TAU	G6P	KG	MAL	HBA	INO
RNFL	-	-	−0.86	−0.84	−0.81	−0.9	−0.83	−0.76	-	-	-	-	-	-	-	-	-
IOP	-	-	0.85	-	-	-	0.74	0.71	-	0.77	-	-	-	-	-	-	-
CDR	−0.86	0.85	-	0.76	0.74	0.83	0.83	-	-	0.7	-	0.71	-	-	-	-	-
TNFα	−0.84	-	0.76	-	0.99	0.93	0.8	0.71	-	-	-	-	-	-	-	-	-
IFNγ	−0.81	-	0.74	0.99	-	0.91	0.77	0.72	-	-	-	-	-	-	-	-	-
TGFβ	−0.9	-	0.83	0.93	0.91	-	0.93	0.91	-	-	-	-	-	-	-	-	-
IL-17A	−0.83	0.74	0.83	0.8	0.77	0.95	-	-	-	0.7	-	-	-	-	-	-	-
ATP	−0.76	0.71	-	0.71	0.72	0.71	-	-	-	0.72	-	-	-	-	-	-	-
LAC	-	-	-	-	-	-	-	-	-	0.72	-	-	-	-	-	-	-
F6P	-	0.77	0.7	-	-	-	0.702	0.72	0.72	-	0.85	-	0.78	-	-	-	-
DSED	-	-	-	-	-	-	-	-	-	0.85	-	-	-	-	-	-	-
TAU	-	-	0.71	-	-	-	-	-	-	-	-	-	-	-	-	-	0.73
G6P	-	-	-	-	-	-	-	-	-	0.78	0.75	0.7	-	0.71	-	0.73	-
KG	-	-	-	-	-	-	-	-	-	-	-	-	-	-	-	0.78	-
MAL	-	-	-	-	-	-	-	-	-	-	-	-	-	-	-	0.79	-
HBA	-	-	-	-	-	-	-	-	-	-	-	-	-	0.78	0.79	-	-

LAC-L, anthranillic acid; F6P, fructose-6-phosphate; DSED, D-sedoheptulose 1,7-phosphate; TAU, taurine; G6P, glucose-6-phosphate; KG, ketoglutarate; MAL, malonate.;HBA, hydroxybutyric acid; INO, inosine.

### 3.7 Integrated biomarker analysis using clinical, ATP, cytokine, and metabolomic profile data identified potential markers that distinguish PACG patients

MetaboAnalyst was used to determine the levels of IOP, CDR, RNFL thickness, ATP, and cytokine levels as well as the levels of different metabolites. The values obtained for IOP, CDR, RNFL thickness, ATP, and cytokines were 1 each for AUC, while those for taurine, fructose-6-phosphate, D-sedoheptulose 1,7-phosphate, and glucose-6-phosphate were 0.928, 0.928, 0.91 and 0.91, respectively. These results show that cytokines, along with metabolites, could be early markers and, concomitant with other parameters, could predict the progression of PACG. The results of the integrated biomarkers analysis are provided in Table 2.

**TABLE 2:** Results of integrated biomarker analysis.

Name	AUC
IOP	1.0
RNFL	1.0
CDR	1.0
ATP	1.0
TNF	1.0
TGF	1.0
IFN	1.0
IL-17A	1.0
Taurine	0.928
Fructose-6-phosphate	0.928
D-sedoheptulose 1,7-phosphate	0.910
Glucose-6-phosphate	0.910

Because microglia are implicated in many neurodegenerative diseases, including glaucoma (Marcin et al., 2005; Pulukool et al., 2021), we used N9 microglia to elucidate the mechanisms associated with TNFα-mediated metabolic remodeling with potential implications for glaucoma.

### 3.8 TNFα treatment induced metabolic remodeling and activation of immuno-metabolism in N9 microglial cells

**FIGURE 3:**
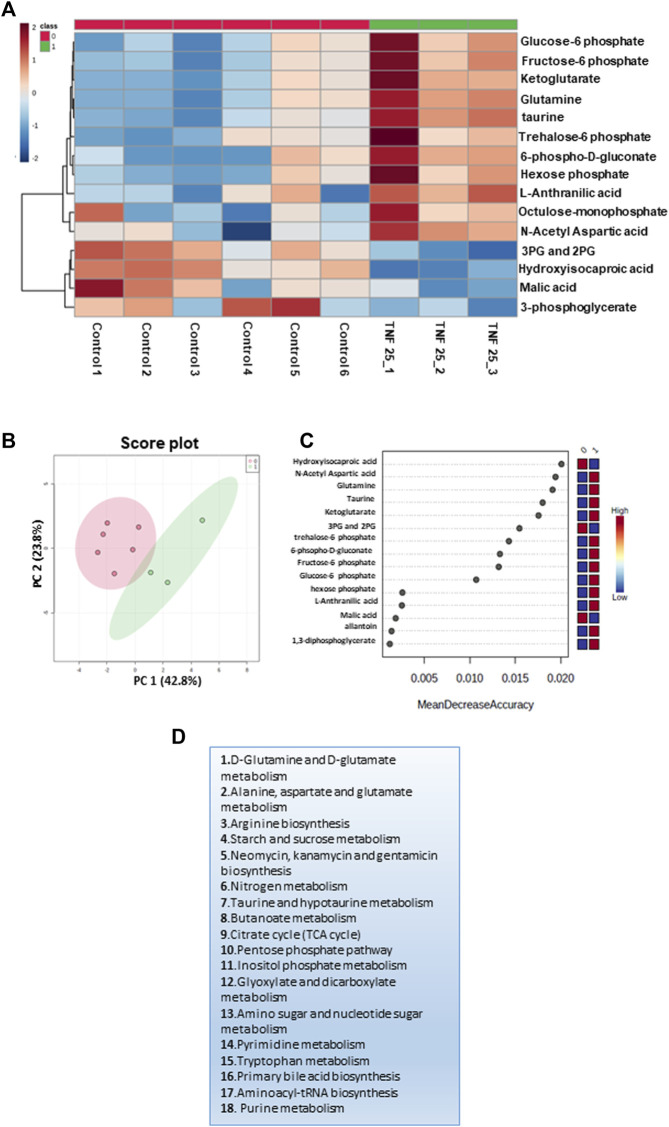
Targeted metabolomics analysis of the cell culture supernatant of N9 cells treated with TNF 25 ng in negative mode metabolomic analysis, showing treatment of N9 cells with TNF (25 ng) in the negative mode. **(A)** Unsupervised heat map representing significantly altered metabolites between control sets and TNFα (25 ng) treated sets in the negative mode. **(B)** Unbiased PCA clustering is shown in the negative mode using a score plot. **(C)** Random forest analysis. **(D)** The pathways to which differential metabolites belong were obtained at an FDR of 0.25 in the negative mode. Panels 3A–C were generated using MetaboAnalyst 5.0 (Version 5.0; URL: https://www.metaboanalyst.ca/).

The inflammatory response is known to induce the macrophage/microglia M1 phenotype (Orihuela et al., 2016), which is associated with increased glycolysis, nucleotide biosynthesis, and reduced mitochondrial electron transport chain function (Orihuela et al., 2016). A potential role of TNFα and ATP is implicated in modulating metabolic remodeling in many cell types, including immune cells (Pulukool et al., 2022). To probe if TNFα-induced metabolic remodeling involved an immuno-metabolism similar to that observed in PACG, we carried out MRM-based targeted metabolic analysis of cell culture supernatant from 25 ng TNFα-treated N9 microglial cells for 12 h. The relative levels of 76 metabolites that were targeted in the negative mode were determined across controls and TNFα-treated N9 cell culture supernatant samples, and 23 metabolites were identified. After normalization of the data sets with internal standards, 18 metabolites with a coefficient of variation (CV) of less than 20% were used for further analysis. Fifteen significant differential metabolites were obtained by analyzing the data for significance (p< 0.05, FDR = 0.25) (Supplementary Figure S5), and the results are represented using a heat map (Figure 3A). The PCA analysis performed on the samples clustered them into two different groups (Supplementary Figure S5). The groups are further represented by the score plot (Figure 3B). The metabolites include glucose-6-phosphate, fructose-6-phosphate, 6-phospho-D-gluconate, glutamine, etc. A random forest analysis showed hydroxyisocaproic acid, N-acetyl aspartate, glutamine, taurine, and ketoglutarate as the metabolites that distinguish TNFα-treated samples from the controls (Figure 3C). The metabolites were grouped into 18 metabolic pathways (Figure 3D). The results demonstrated the role of TNFα-induced metabolic remodeling in N9 microglia *in vitro*, which might have potential implications in PACG. The results reiterate a potential involvement of microglia and immuno-metabolism in the disease process.

### 3.9 Comparative analysis of metabolic pathways obtained for PACG and TNFα- or ATP-treated microglial cell culture supernatant shows overlapping pathways

**FIGURE 4:**
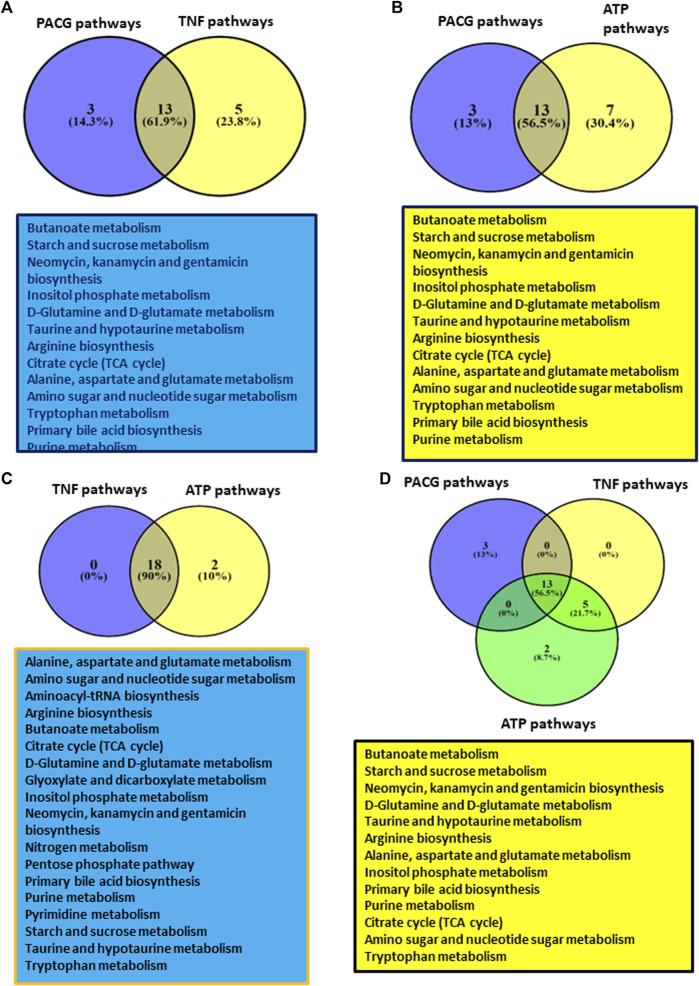
Showing results of comparative analysis of metabolic pathways among PACG with TNFα or ATP: **(A)** Comparative analysis of PACG with TNFα showed 13 common pathways. **(B)** Comparative analysis of PACG with the ATP-treated microglial cell culture supernatant showed 13 common pathways. **(C)** Metabolic pathway data on TNFα-treated microglia completely overlap with those of ATP-treated microglial data sets. **(D)** Comparative analysis of PACG with ATP and TNFα showed 13 common pathways. Panels 4A–D were created using VENNY2.1 (Version 2.1; URL: https://bioinfogp.cnb.csic.es/tools/venny/).

Comparative analysis of metabolic pathways obtained for PACG and TNFα showed 13 common pathways (Figure 4A). These pathways include butanoate; the starch and sucrose metabolism; the inositol phosphate metabolism; metabolism of various amino acids like glutamine, glutamate, arginine, alanine, aspartate, and tryptophan, etc.; the purine metabolism, etc. Because ATP was elevated in the PACG patient cohort, we also compared the metabolic pathways of the PACG patients with those of the ATP-treated microglial cell culture supernatant from our previous study (Pulukool et al., 2021). Thirteen metabolic pathways (Figure 4B) were common between the PACG patients and the ATP-treated microglial culture supernatant. Furthermore, a comparison of the metabolic pathway data of TNFα-treated microglia with that of ATP-treated microglial data showed a complete overlap (Figure 4C). Comparative analysis of PACG data with ATP and TNFα results showed 13 common pathways (Figure 4D). Taken together, these results show an association of immuno-metabolism with PACG potentially involving microglia. The results also suggest a potential role for elevated ATP and TNFα in microglial inflammation with implications for PACG.

Overall, our results indicate clinical parameters characteristic of PACG, elevated levels of ATP and inflammatory cytokines (TNFα, TGFβ, IFNγ, and IL-17A) in the aqueous humor and plasma, respectively, as well as metabolic remodeling involving immuno-metabolism in the aqueous humor. Mechanistic validation through cell culture studies using N9 microglia reveals that TNFα-modulated metabolic remodeling involves immuno-metabolites, which significantly overlaps with the metabolic profile of aqueous humor from PACG. Taken together, the results show elevated ATP and cytokines and a potential role for TNFα-induced metabolic remodeling in microglia involving immuno-metabolism with implications for PACG.

## 4 Discussion

Our PACG patient cohort exhibited increased IOP and CDR with reduced RNFL thickness, which are the characteristic features of PACG. Elevated ATP levels in aqueous humor are observed in the PACG patient group. Elevated ATP levels in aqueous and vitreous humor are associated with many forms of glaucoma (Pulukool et al., 2021). ATP signaling is implicated in aqueous humor draining and inflammation (Pulukool et al., 2021). Hence, the high IOP might invoke a microglial inflammatory response through ATP signaling. Furthermore, ATP levels strongly correlate with TNFα, IFNγ, and TGFβ. Our previous study shows ATP invoked expression of TNFα, IFNγ, and TGFβ in N9 microglia (Pulukool et al., 2021). ATP levels showed a strong negative correlation with RNFL thickness.

Elevated levels of TNFα, IFNγ, IL17A, and TGFβ are observed in PACG, which is in alignment with previous reports on glaucoma (Wang et al., 2018). TGFβ was shown to induce ATP secretion in cancer cells (Takai et al., 2012). IL17 is shown to be associated with many retinal degenerative diseases, displays a differential effect on retinal astrocytes and retinal pigment epithelial cells, and also damages the blood–brain barrier (Zhong and Sun, 2022). IFNγ was shown to induce RGC death, and its suppression was neuroprotective (Husain et al., 2014). Although TGFβ is essential for the protection of neurons in the retina, it is also shown to be associated with the expression of vascular endothelial growth factor (VEGF), leading to neovascularization and fibrosis around new vessels as well as remodeling of extracellular matrix in the trabecular network (Verrecchia and Mauviel, 2007). Consistent with this, our correlation analysis shows a strong negative correlation of RNFL thickness with TNFα, IFNγ, IL17A, and TGFβ. Hence, elevated cytokines might invoke an inflammatory response, which might have potential consequences for PACG.

Targeted metabolomic analysis of our patient cohort shows changes in the pentose phosphate pathway and the sucrose, branch chain amino acid, and nucleotide metabolisms, etc. Significantly elevated ATP levels, glucose-6-phosphate, fructose-6-phosphate, etc., in the aqueous humor of PACG were demonstrated in our study. The metabolic profile in this study shows elevated glycolysis and nucleotide biosynthesis and is characteristic of the inflammatory response. Previous studies have shown that elevated lactate and succinate are imperative for cytokine secretion (Lauro and Limatola, 2020). Immune cells like microglia, macrophages, or T cells show an M1 phenotype that exhibits upregulation of glycolysis and nucleotide biosynthesis with reduced mitochondrial activity during inflammation (Pulukool et al., 2021). Indeed, random forest analysis identified metabolites like glucose-6-phosphate and fructose-6-phosphate that distinguish PACG from the control.

Metabolomic analysis shows glucuronic acid as one of the elevated metabolites in PACG. Interestingly, fructose-6-phosphate strongly correlated with ATP and IL17A levels in the present study. Previous studies have also demonstrated that IL17 induces immuno-metabolism in immune cells (Bechara et al., 2021). Studies have shown that glucuronic acid activates TLR4 and invokes ROS (Lewis et al., 2013), while inosine and taurine are neuroprotective, which has implications for disease. Malonate, which is elevated in our PACG patient cohort, was previously shown to induce cell death by collapsing the mitochondrial potential, invoking ROS.

Elevated hydroxybutyrate levels were found in the aqueous humor of PACG (Brown et al., 1986). Studies in a mouse model of normal tension glaucoma have shown that every alternative day fasting leads to elevated levels of beta-hydroxybutyrate. Increased HBA leads to increased histone acetylation and upregulation of neurotrophic factors and catalase in the retina. Alpha-ketoglutarate, which is elevated in the aqueous humor of PACG, was found to be involved in the polarization of macrophage into the M2 phenotype and exert an anti-inflammatory response.

The details of these metabolite-mediated effects are provided in Supplementary Table S3. Previous studies have demonstrated the involvement of cytokines and ATP with inflammation and immuno-metabolism (Pulukool et al., 2021). Our results show activation of the tryptophan metabolism in PACG. Metabolites belonging to the tryptophan pathway induce neuronal cell death (Lauro and Limatola, 2020) with implications for PACG. Taken together, the results of metabolomic analysis show an inflammatory phenotype and a response to mitigate the effect by a compensatory metabolism.

Our commonality analysis using pathways obtained from the analysis of PACG proteomic and rat model metabolomics data sets from published literature showed significant overlapping pathways. The hyaluronic acid-injected rat model of glaucoma is representative of PACG (Moreno et al., 2005; Bouhenni et al., 2012). The metabolomics pathways from these sets demonstrate that the pathways are highly conserved across taxa, populations, study settings, and techniques used. The comparative analysis with other data sets from published literature could help mitigate the disadvantages of low sample numbers reported in our study. Furthermore, metabolomic analysis of amino acids and carnitine of aqueous humor from PACG patients showed deregulation of arginine levels (Zhang et al., 2023). The results from our study reiterates that deregulated pathways have a potential role in disease progression. It also shows that the metabolomics analysis could show representative deregulated pathways associated with PACG.

The clinical analysis of elevated levels of ATP and cytokines and the metabolic profile in our study is indicative of an inflammatory milieu, which is found to be associated with the disease (Pulukool et al., 2021). Microglia, which are immune cells of the CNS and retina, are implicated in glaucoma (Pulukool et al., 2021). Consistent with this, activated microglia are associated with glaucoma (Pulukool et al., 2021). Immune cells, including microglia, exhibit a shift to aerobic glycolysis during inflammation (Pulukool et al., 2021). The increased glycolysis, pentose phosphate pathway, and nucleotide biosynthesis are found to be essential for growth and proliferation (Lauro and Limatola, 2020). Our metabolomic analysis on cell culture supernatant from TNFα-treated microglial cells shows elevated levels of metabolites belonging to the alanine, aspartate, and glutamate metabolism, the glutamine and glutamate metabolism, the tryptophan metabolism, the TCA cycle, the amino sugar and nucleotide sugar metabolism, and the pentose phosphate pathway. Studies have shown that inhibition of glycolysis by a non-degradable analog of glucose impairs inflammatory response (Wang et al., 2014). Glucose-6-phosphate and fructose-6-phosphate are found to be elevated in microglia. Upregulation of glycolysis resulting in the M1 phenotype of microglia and other immune cells is reported during activation (Wang et al., 2014). Previous studies have also shown the upregulation of glycolysis by TNFα in many cancer cell types, endothelial cells, and immune cells (Vaughan et al., 2013). Activation of tryptophan metabolic pathways is also observed in an *in vitro* cell culture study with implications for glaucoma (Pulukool et al., 2021).

Interestingly, glyceraldehyde-3-phosphate/-2-phosphate and 1,3-bisphosphoglycerate were found to be very low in TNFα-treated data sets. Previous studies have shown that treatment with TNFα will temporarily inhibit GAPDH to protect it from ROS (Bereta and Bereta, 1995). The glutamine metabolism is upregulated in microglia during an immune response, resulting in M1 polarization (Viola et al., 2019). Similarly, in our study, alpha-ketoglutarate, which is a downstream metabolite of glutaminolysis, is elevated in microglia treated with TNFα. Taurine, a neuroprotective metabolite (Jafri et al., 2019), was also found to be elevated in TNFα-treated microglia.

Overall, our results show that TNFα treatment in N9 microglia invokes immuno-metabolism with potential implications for PACG. Our previous studies show immuno-metabolism is involved in ATP-treated microglia (Pulukool et al., 2021). Consistent with this observation, the metabolic pathway profile of the TNFα-treated microglial cell culture supernatant overlapped with that of the ATP-treated microglial cell culture supernatant. Similarly, extracellular ATP acts as an inflammatory molecule that not only induces cytokines but also induces metabolic remodeling (Pulukool et al., 2021). Interestingly, the TNFα-induced inflammatory response is dependent on P2 receptors (Pulukool et al., 2022). Our results from an *in vitro* N9 cell culture model system suggest a role for TNFα and ATP in metabolic remodeling with potential implications for PACG.

Our analysis indicates that elevated ATP and cytokines and metabolic remodeling involving immuno-metabolites are associated with PACG. TNFα is implicated in inducing metabolic remodeling involving immuno-metabolism. There is a significant overlap of metabolic pathways between PACG data and TNFα- or ATP-treated N9 microglial cell culture supernatant data sets. To our knowledge, this might be the first report showing an association of elevated levels of ATP in aqueous humor, plasma cytokine levels, and activation of immuno-metabolism with PACG. TNFα-induced metabolic remodeling involving immuno-metabolism in microglia *in vitro* has potential implications for the disease process. Our findings suggest opportunities for future studies to validate the levels of aqueous humor ATP, plasma cytokines, and metabolites as potential biomarkers. These findings could be validated on a larger cohort of PACG patients.

These results suggest ATP, cytokines, and metabolites belong to the immuno-metabolism and tryptophan metabolism groups as biomarkers in PACG. The results also suggest the potential of using purinergic signaling inhibitors, anti-TNFα/IFNγ antibodies, and inhibitors of IDO1/2 as potential therapeutic targets that might aid in better management of PACG. The results from the present study not only helped to identify the molecular signature and mechanistic aspects associated with PACG but also potential biomarkers and therapeutic targets, which will aid in the better management of the disease.

## 5 Conclusions

In our cohort of patients with PACG, we observed elevated IOP and CDR, increased levels of ATP in aqueous humor, elevated cytokines in plasma, and reduced RNFL thickness. Metabolic remodeling involving immuno-metabolism is associated with PACG. Studies with N9 microglia show that TNFα-induced metabolic rewiring involves immuno-metabolism. Comparative analysis of metabolic pathways obtained for PACG with TNFα- and ATP-treated N9 microglia cell culture supernatant shows a considerable overlap of pathways. This study could be the first thorough analysis to investigate the connection between high levels of ATP, cytokines, and metabolic changes related to the immune metabolism in PACG. Mechanistically, our work shows a potential role for TNFα- and ATP-mediated metabolic rewiring involving immuno-metabolism and microglial inflammation with the disease process. ATP, cytokines, taurine, fructose-6-phosphate, glucose-6-phosphate, and D-sedoheptulose 1,7 phosphate could be potential biomarkers in PACG. TNFα and purinergic signaling could be potential therapeutic targets in the disease.

## Data Availability

The original contributions presented in the study are included in the article/Supplementary Material; further inquiries can be directed to the corresponding author.
