# Supplementary material for: Integrated clinical and metabolomic analysis identifies molecular signatures, biomarkers, and therapeutic targets in primary angle closure glaucoma
Source: Front Mol Biosci. 2024 Aug 9;11:1421030. doi: 10.3389/fmolb.2024.1421030 (PMC11341363; doi:10.3389/fmolb.2024.1421030)
Supplement: Supplementary file 1 [file Image5.pdf]

Supplementary Figure 5a: Pathways obtained from differential metabolites from Hyaluronate induced rat model

1. Nitrogen metabolism
2. Alanine, aspartate and glutamate metabolism
3. Glyoxylate and dicarboxylate metabolism
4. Arginine and proline metabolism
5. Arginine biosynthesis
6. Butanoate metabolism
7. Valine, leucine and isoleucine degradation
8. Valine, leucine and isoleucine biosynthesis
9. Biotin metabolism
10. Fructose and mannose metabolism
11. Pantothenate and CoA biosynthesis
12. Citrate cycle (TCA cycle)
13. Glycolysis / Gluconeogenesis
14. Galactose metabolism
15. Glutathione metabolism
16. Lysine degradation
17. Porphyrin metabolism
18. Glycine, serine and threonine metabolism
19. Pyrimidine metabolism
20. Steroid biosynthesis
21. Tyrosine metabolism
22. Amino sugar and nucleotide sugar metabolism
23. Primary bile acid biosynthesis
24. Purine metabolism
25. Steroid hormone biosynthesis
26. Histidine metabolism

Supplementary Figure 5b: Pathways obtained from proteomic data set of PACG using significant differential proteins from literature.

1. Systemic lupus erythematosus
2. Neutrophil extracellular trap formation
3. Complement and coagulation cascades
4. Viral carcinogenesis
5. Alcoholism
6. Cholesterol metabolism
7. Amino sugar and nucleotide sugar metabolism
8. AGE-RAGE signaling pathway in diabetic complications
9. Amoebiasis
10. Regulation of actin cytoskeleton
11. Viral myocarditis
12. Fructose and mannose metabolism
13. Leishmaniasis
14. Spliceosome
15. Focal adhesion
16. Human papillomavirus infection
17. ECM-receptor interaction
18. Epithelial cell signaling in Helicobacter pylori infection
19. Prolactin signaling pathway
20. Proteoglycans in cancer
21. Protein digestion and absorption
22. Phagosome
23. Relaxin signaling pathway
24. Tuberculosis
25. Galactose metabolism
26. Acute myeloid leukemia
27. Human immunodeficiency virus 1 infection
28. Yersinia infection
29. Th1 and Th2 cell differentiation
30. Staphylococcus aureus infection
31. Chronic myeloid leukemia
32. Diabetic cardiomyopathy
33. Antigen processing and presentation
34. T cell receptor signaling pathway
35. Th17 cell differentiation
36. Human T-cell leukemia virus 1 infection
37. PD-L1 expression and PD-1 checkpoint pathway in cancer
38. Leukocyte transendothelial migration
39. Sphingolipid signaling pathway

**40. Pertussis**
**41. RNA transport**
**42. Other glycan degradation**
**43. Estrogen signaling pathway**
**44. Lipid and atherosclerosis**
**45. Lysosome**
**46. Legionellosis**
**47. Vitamin digestion and absorption**
**48. Phototransduction**
**49. Pentose phosphate pathway**
**50. Glycolysis / Gluconeogenesis**
**51. Salivary secretion**
**52. Prion disease**
**53. Longevity regulating pathway**
**54. Renin secretion**
**55. Parkinson disease**
**56. Gastric acid secretion**
**57. Oocyte meiosis**
**58. Vascular smooth muscle contraction**
**59. African trypanosomiasis**
**60. Fluid shear stress and atherosclerosis**
**61. Pathways of neurodegeneration**
**62. Aldosterone synthesis and secretion**
**63. Inflammatory mediator regulation of TRP channels**
**64. Glucagon signaling pathway**
**65. HIF-1 signaling pathway**
**66. Neurotrophin signaling pathway**
**67. Long-term potentiation**
**68. Amphetamine addiction**
**69. Apelin signaling pathway**
**70. PPAR signaling pathway**
**71. Thyroid hormone synthesis**
**72. Glioma**
