# Supplementary material for: Integrated clinical and metabolomic analysis identifies molecular signatures, biomarkers, and therapeutic targets in primary angle closure glaucoma
Source: Front Mol Biosci. 2024 Aug 9;11:1421030. doi: 10.3389/fmolb.2024.1421030 (PMC11341363; doi:10.3389/fmolb.2024.1421030)
Supplement: Supplementary file 2 [file Table1.pdf]

MRM Details

Compound Name	ISTD	Precursor ion	Product ion	Dwell	Frag (V)	CE (V)	Cell Acc (V)	Polarity
MALTOTETRAOSE	No	665.2	161.1	20	380	13	5	Negative
NAD+_neg	No	662	540	20	380	21	4	Negative
glutathione disulfide_neg	No	611	306	20	380	30	4	Negative
UDP-N-acetyl-glucosamine	No	606	385	20	380	28	4	Negative
UDP-D-glucuronate	No	579	403	20	380	26	4	Negative
UDP-D-glucose	No	565	323	20	380	25	4	Negative
cholesteryl sulfate	No	465.2	97	20	380	39	4	Negative
dGDP_neg	No	426.12	159	20	380	27	4	Negative
ADP_neg	No	426.1	159	20	380	27	4	Negative
trehalose-6-Phosphate	No	421	79	20	380	36	4	Negative
Cholestrol	No	385.3	217.7	20	380	8	5	Negative
Farnesyl-PP	No	381	189.7	20	380	40	5	Negative
Farnesyl-PP	No	381	78.7	20	380	40	5	Negative
L-Gibberilic acid	No	345	143	20	380	29	2	Negative
L-Gibberilic acid	Yes	345	143	3	380	5	2	Negative
Maltose	No	341.1	161	20	380	5	5	Negative
Maltose	No	341.1	73	20	380	21	5	Negative
FBP/GBP	No	338.9	96.8	20	380	15	5	Negative
FBP/GBP	No	338.9	79	20	380	60	5	Negative
octulose-monophosphate (O8P-O1P)	No	319	97	20	380	22	4	Negative
Eicosatetraenoic acid-D8 (ETA)	Yes	311.5	245.194	3	380	20	2	Negative
Eicasatetraenoic acid-D8 (ETA)	Yes	311.5	59.05	3	380	35	2	Negative
D-sedoheptulose-1-7-phosphate	No	289	97	20	380	27	4	Negative
Stearic acid	No	283.48	265.2	20	380	40	2	Negative
Stearic acid	No	283.48	45	20	380	40	2	Negative
6-phospho-D-gluconate	No	275	97	20	380	13	4	Negative
inosine	No	267	135	20	380	27	4	Negative
S-ribosyl-L-homocysteine_neg	No	266	134	20	380	20	4	Negative
1,3-diphopshateglycerate	No	265	79	20	380	37	4	Negative
L-palmitic acid	No	264	155.8	20	380	15	2	Negative
L-palmitic acid	No	264	146	20	380	15	2	Negative

Frctose-6-Phosphate	No	259	97.1	20	380	5	5	Negative
Glucose-6-Phosphate	No	259	97	20	380	5	5	Negative
Frctose-6-Phosphate	No	259	79.1	20	380	37	5	Negative
hexose-phosphate	No	259	79	20	380	42	4	Negative
uridine	No	243	200	20	380	21	4	Negative
Tetradecanoic acid-D3 (TDA-D3)	Yes	230.3	212.2	3	380	40	2	Negative
Tetradecanoic acid-D3 (TDA-D3)	Yes	230.3	59	3	380	40	2	Negative
Tetradecanoic acid-D3 (TDA-D3)	Yes	230.3	58	3	380	40	2	Negative
Ribose-5-phosphate	No	229.01	138.98	20	380	15	5	Negative
Ribose-5-phosphate	No	229.01	96.97	20	380	15	5	Negative
L-Zeatine	No	218	173	20	380	10	2	Negative
Zeatine	Yes	218	173	3	380	10	2	Negative
L-Zeatine	No	218	133.5	20	380	21	2	Negative
L-Jasmonic acid	No	209	126.9	20	380	10	2	Negative
L-Jasmonic acid	Yes	209	126.9	3	380	10	2	Negative
L-Jasmonic acid	No	209	59	20	380	9	2	Negative
L-Jasmonic acid	Yes	209	59	3	380	10	2	Negative
L-Tryptophan	No	205	117	20	380	13	2	Negative
L-Tryptophan	Yes	205	117	3	380	10	2	Negative
Citrate-13c6	Yes	197	90	3	380	20	2	Negative
Glucuronic acid	No	193	113	20	380	5	5	Negative
Glucuronic acid	No	193	73.1	20	380	9	5	Negative
3-phosphoglycerate	No	185	97	20	380	17	4	Negative
Glucose-13C6	Yes	185	74	3	380	7	2	Negative
Glucose-13C6	Yes	185	60.8	3	380	20	2	Negative
3PG and 2PG	No	184.9	97	20	380	13	5	Negative
N-acety AA-d3	Yes	177	132.7	20	380	6	2	Negative
N-acety AA-d3	Yes	177	90.8	20	380	6	2	Negative
N-Aetyl Aspartic acid	No	174	88.1	20	380	13	5	Negative
shikimate	No	173	93	20	380	20	4	Negative
sn-glycerol-3-phosphate	No	171	79	20	380	15	4	Negative
Uric acid	No	167.001	124	20	380	17	4	Negative
PEP	No	167	79	20	380	9	5	Negative
PEP	No	167	63	20	380	60	5	Negative
allantoin	No	157.05	114	20	380	17	4	Negative
dihydroorotate	No	157	113	20	380	14	4	Negative

2,3-dihydroxybenzoic acid	No	153	109	20	380	19	4	Negative
L-Glutamic acid D5	No	151	107	20	380	13	2	Negative
Glutamic acid-D5	Yes	151	107	3	380	5	2	Negative
Glutamic acid D5	Yes	151	107	3	380	13	2	Negative
Glutamine-15N2	Yes	147	111	3	380	9	2	Negative
Glutamine	No	145	109	20	380	9	5	Negative
Ketoglutarate	No	145	101.1	20	380	5	5	Negative
Glutamine	No	145	84	20	380	13	5	Negative
Ketoglutarate	No	145	57.1	20	380	20	5	Negative
Octanoic acid	No	143.1	125.1	20	380	20	2	Negative
acetylphosphate	No	139	79	20	380	24	4	Negative
L-Anthranilic aicd	No	137	93.1	20	380	15	2	Negative
L-Anthranilic aicd	No	137	65.2	20	380	20	2	Negative
p-aminobenzoate	No	136.05	92	20	380	18	4	Negative
anthranilate	No	136	92	20	380	18	4	Negative
Malic acid	No	133	115.1	20	380	5	5	Negative
Malic acid	No	133	71.1	20	380	9	5	Negative
Hydroxyisocaproic acid	No	131.006	85.1	20	380	16	4	Negative
taurine	No	124	80	20	380	18	4	Negative
Fumarate	Yes	119	74	3	380	5	2	Negative
Succinic acid	No	117	99.9	20	380	10	5	Negative
succinate	No	117	73	20	380	12	4	Negative
Fumaric acid	No	115	71.1	20	380	5	5	Negative
Fumaric acid	No	115	27.2	20	380	5	5	Negative
Malonate (Propanedioate)	No	103.1	59.1	20	380	5	5	Negative
Malonate (Propanedioate)	No	103.1	41.1	20	380	29	5	Negative
HBA	No	103	59.1	20	380	5	5	Negative
HBA	No	103	41.1	20	380	25	5	Negative
Pyruvate	Yes	89	44	3	380	10	2	Negative
Pyruvate	Yes	89	44	3	380	10	2	Negative
lactate	No	89	43.2	20	380	16	4	Negative
glycolate	No	75	45.2	20	380	13	4	Negative

Source Parameters

Parameter	Value (+)	Value (-)
Gas Temp (°C)	250	250
Gas Flow (l/min)	14	14
Nebulizer (psi)	20	20
SheathGasHeater	350	350
SheathGasFlow	11	11
Capillary (V)	3000	3500
VCharging	1000	1000

Ion Funnel Parameters

Pos High Pressure RF	150
Pos Low Pressure RF	60
Neg High Pressure RF	150
Neg Low Pressure RF	60

Auxiliary

Draw Speed	100.0 µL/min
Eject Speed	100.0 µL/min
Draw Position Offset	0.0 mm
Wait Time After Drawing	2.0 s
Sample Flush Out Factor	5.0
Vial/Well bottom sensing	Yes

Timetable

	Time	Function	Parameter
1	3.00 min	Change Solvent Composition	Solvent composition A: 70.00 % B:30.00 %
2	3.00 min	Change Flow	Flow: 0.3 mL/min
3	12.00 min	Change Solvent Composition	Solvent composition A: 98.00 % B:2.00 %
4	12.00 min	Change Flow	Flow: 0.3 mL/min
5	15.00 min	Change Solvent Composition	Solvent composition A: 98.00 % B:2.00 %
6	15.00 min	Change Flow	Flow: 0.3 mL/min
7	16.00 min	Change Solvent Composition	Solvent composition A: 15.00 % B:85.00 %
8	16.00 min	Change Flow	Flow: 0.3 mL/min
9	23.00 min	Change Solvent Composition	Solvent composition A: 15.00 % B:85.00 %
10	23.00 min	Change Flow	Flow: 0.3 mL/min

Solvent Composition

	Channel	Ch. 1 Solv.	Name 1	Ch2 Solv.	Name 2	Selected	Used	Percent
1	A	100.0 % Water V.03	pH 9 95% water 5%ACN	100.0 % Water V.03	pH 9 95% water 5%ACN	Ch. 2	Yes	15.00 %
2	B	100.0 % Acetonitrile V.03	100% ACN	100.0 % Acetonitrile V.03	100% ACN	Ch. 2	Yes	85.00 %
