# Supplementary material for: Integrated clinical and metabolomic analysis identifies molecular signatures, biomarkers, and therapeutic targets in primary angle closure glaucoma
Source: Front Mol Biosci. 2024 Aug 9;11:1421030. doi: 10.3389/fmolb.2024.1421030 (PMC11341363; doi:10.3389/fmolb.2024.1421030)
Supplement: Supplementary file 3 [file Table2.pdf]

	RNFL	IOP	CDR	TNFα	IFNγ	TGFβ	IL-17A	ATP	LAC	F6P	DSED	TAU	G6P	KG	MAL	HBA	INO
RNFL			-0.86	-0.84	-0.81	-0.9	-0.83	-0.76									
IOP			0.85				0.74	0.71		0.77							
CDR	-0.86	0.85		0.76	0.74	0.83	0.83			0.7		0.71					
TNFα	-0.84		0.76		0.99	0.93	0.8	0.71									
IFNγ	-0.81		0.74	0.99		0.91	0.77	0.72									
TGFβ	-0.9		0.83	0.93	0.91		0.93	0.91									
IL-17A	-0.83	0.74	0.83	0.8	0.77	0.95				0.7							
ATP	-0.76	0.71		0.71	0.72	0.71				0.72							
LAC										0.72							
F6P		0.77	0.7				0.702	0.72	0.72		0.85		0.78				
DSED										0.85							
TAU			0.71														0.73
G6P										0.78	0.75	0.7		0.71		0.73	
KG																0.78	
MAL																0.79	
HBA														0.78	0.79		

Supplementary Table 2- The result of significant differential metabolites using Pearson's correlation analysis

F6P- Fructose 6 phosphate
DSED- Dsedoheptulose1,7-Phosphate
TAU- Taurine
G6P- glucose 6 phosphate
KG- Ketoglutarate
MAL- Malonate
HBA- Hydrosxybutyric acid
INO- inosine
LAC- L- Anthranillic acid
