# Supplementary material for: Integrated clinical and metabolomic analysis identifies molecular signatures, biomarkers, and therapeutic targets in primary angle closure glaucoma
Source: Front Mol Biosci. 2024 Aug 9;11:1421030. doi: 10.3389/fmolb.2024.1421030 (PMC11341363; doi:10.3389/fmolb.2024.1421030)
Supplement: Supplementary file 4 [file Image4.pdf]

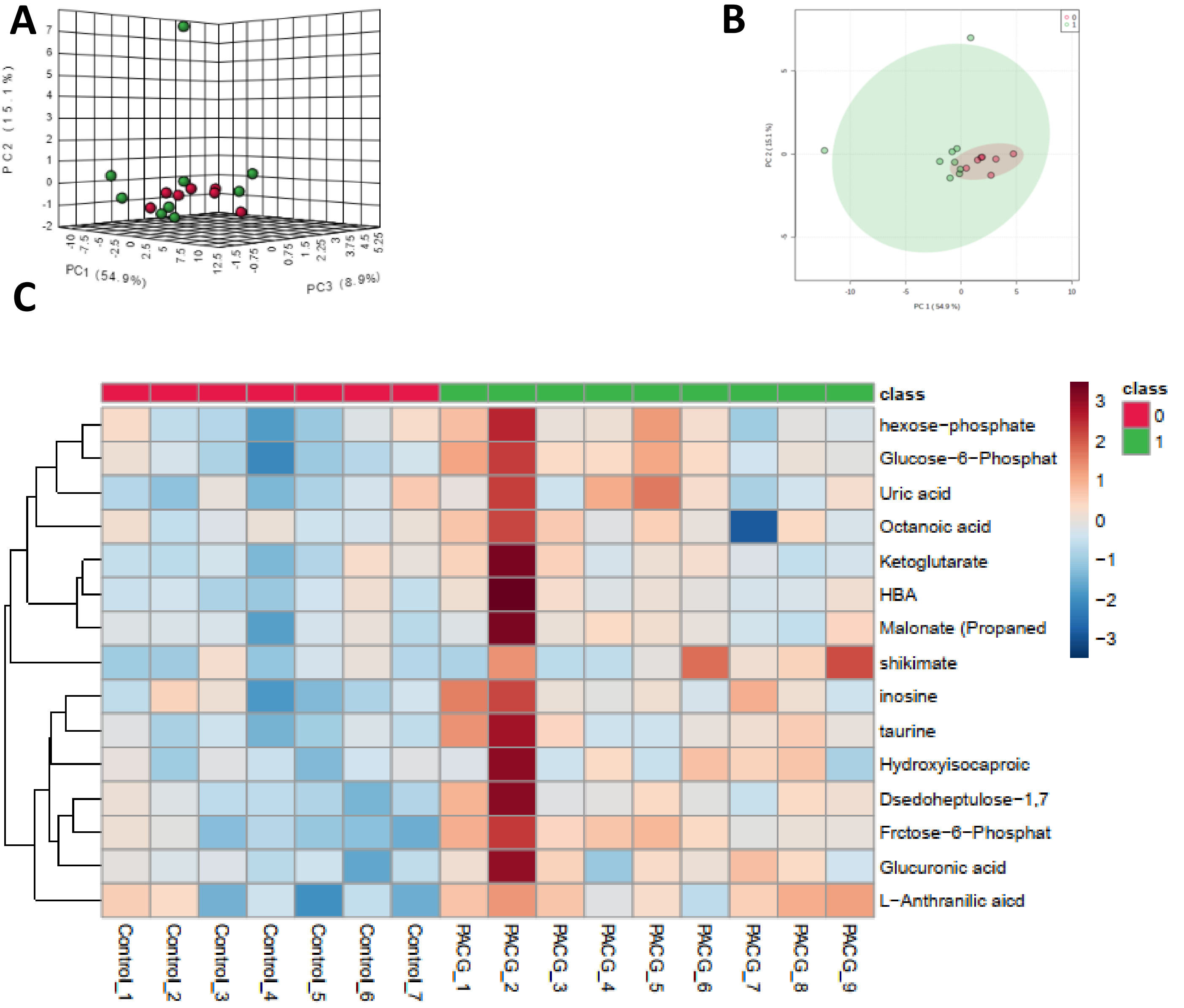

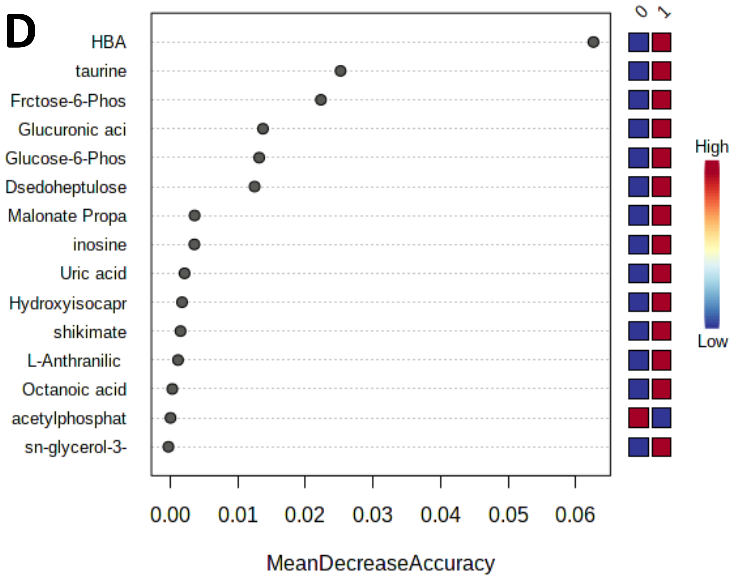

Supplementary Figure 4:

A) PCA plot of the control (red color) and PACG cohort(Green color)

B) Showing score plot of control and PACG cohort

C) Heat map of metabolomic analysis of control and PACG group.

D) Random forest analysis distinguishing control and PACG

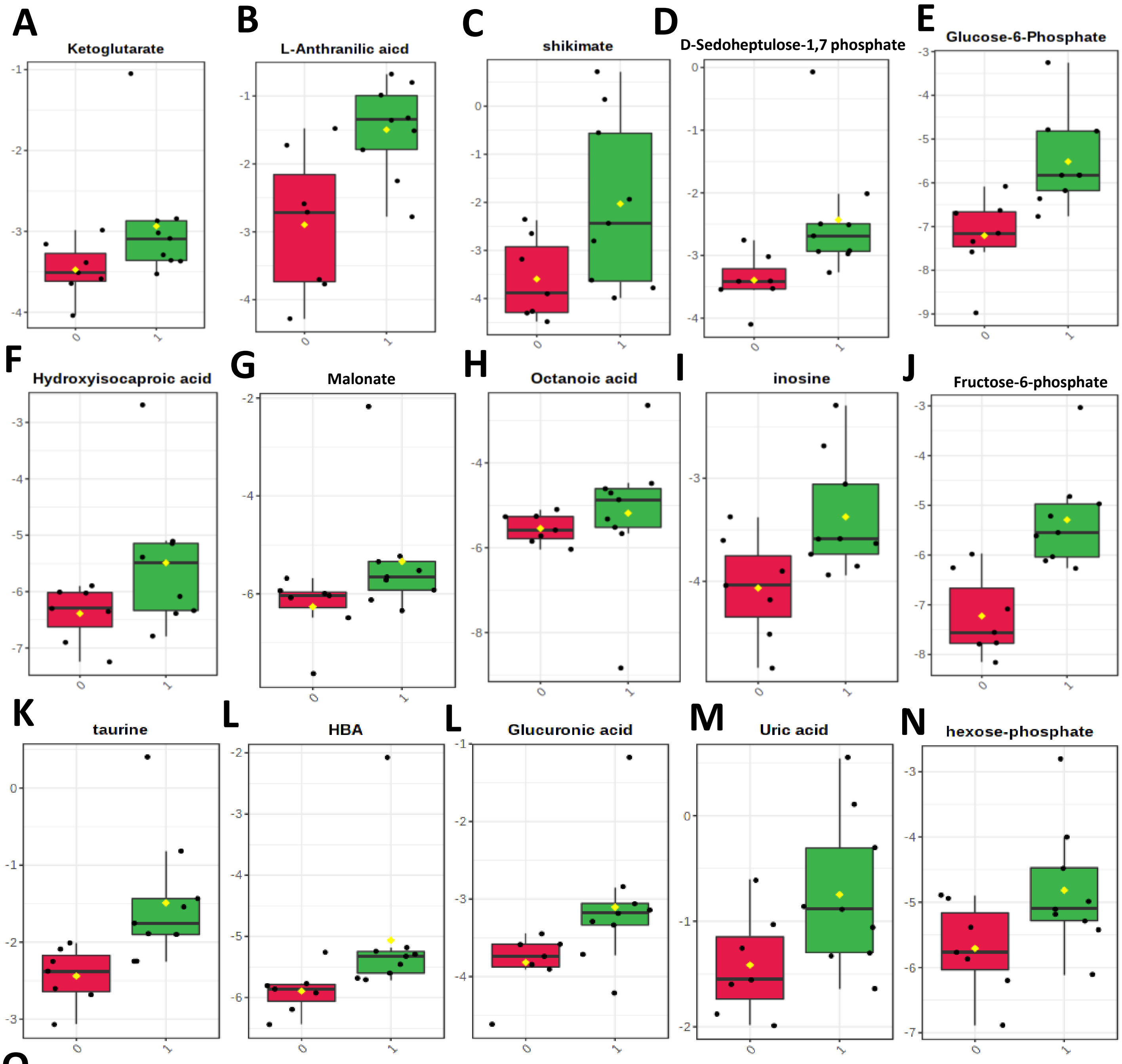

O

Butanoate metabolism
Starch and sucrose metabolism
Neomycin, kanamycin and gentamicin biosynthesis
Inositol phosphate metabolism
Synthesis and degradation of ketone bodies
D-Glutamine and D-glutamate metabolism
Taurine and hypotaurine metabolism
Ascorbate and aldarate metabolism
Purine metabolism
Arginine biosynthesis
Pentose and glucuronate interconversions
Citrate cycle (TCA cycle)
Alanine, aspartate and glutamate metabolism
Amino sugar and nucleotide sugar metabolism
Tryptophan metabolism
Primary bile acid biosynthesis

0- control

1- PACG

Supplementary Figure. 4: **A-N)** Showing 15 significant metabolites in the aqueous humor of PACG patients compared to control. **O**) List of pathways to which significant differential metabolites
