# Supplementary material for: Integrated clinical and metabolomic analysis identifies molecular signatures, biomarkers, and therapeutic targets in primary angle closure glaucoma
Source: Front Mol Biosci. 2024 Aug 9;11:1421030. doi: 10.3389/fmolb.2024.1421030 (PMC11341363; doi:10.3389/fmolb.2024.1421030)
Supplement: Supplementary file 5 [file Image2.pdf]

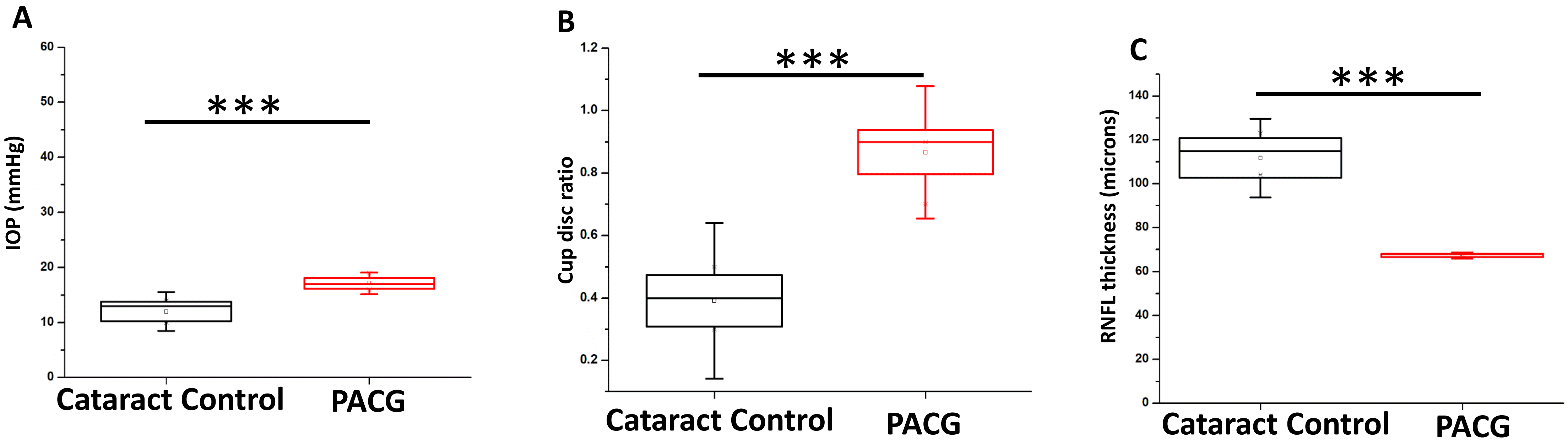

Supplementary Figure 2: Showing assessment of retrospective clinical parameters **A.** The IOP profile in PACG group (n=6) compared to cataract control (n=8). **B.** The cup disc ratio in PACG group (n=9) compared to cataract control (n=9). **C.** Retinal nerve fiber layer thickness (RNFL) thickness in PACG patients (n=2) compared to cataract control eye (n=4).
