# Supplementary material for: Integrated clinical and metabolomic analysis identifies molecular signatures, biomarkers, and therapeutic targets in primary angle closure glaucoma
Source: Front Mol Biosci. 2024 Aug 9;11:1421030. doi: 10.3389/fmolb.2024.1421030 (PMC11341363; doi:10.3389/fmolb.2024.1421030)
Supplement: Supplementary file 6 [file Image3.pdf]

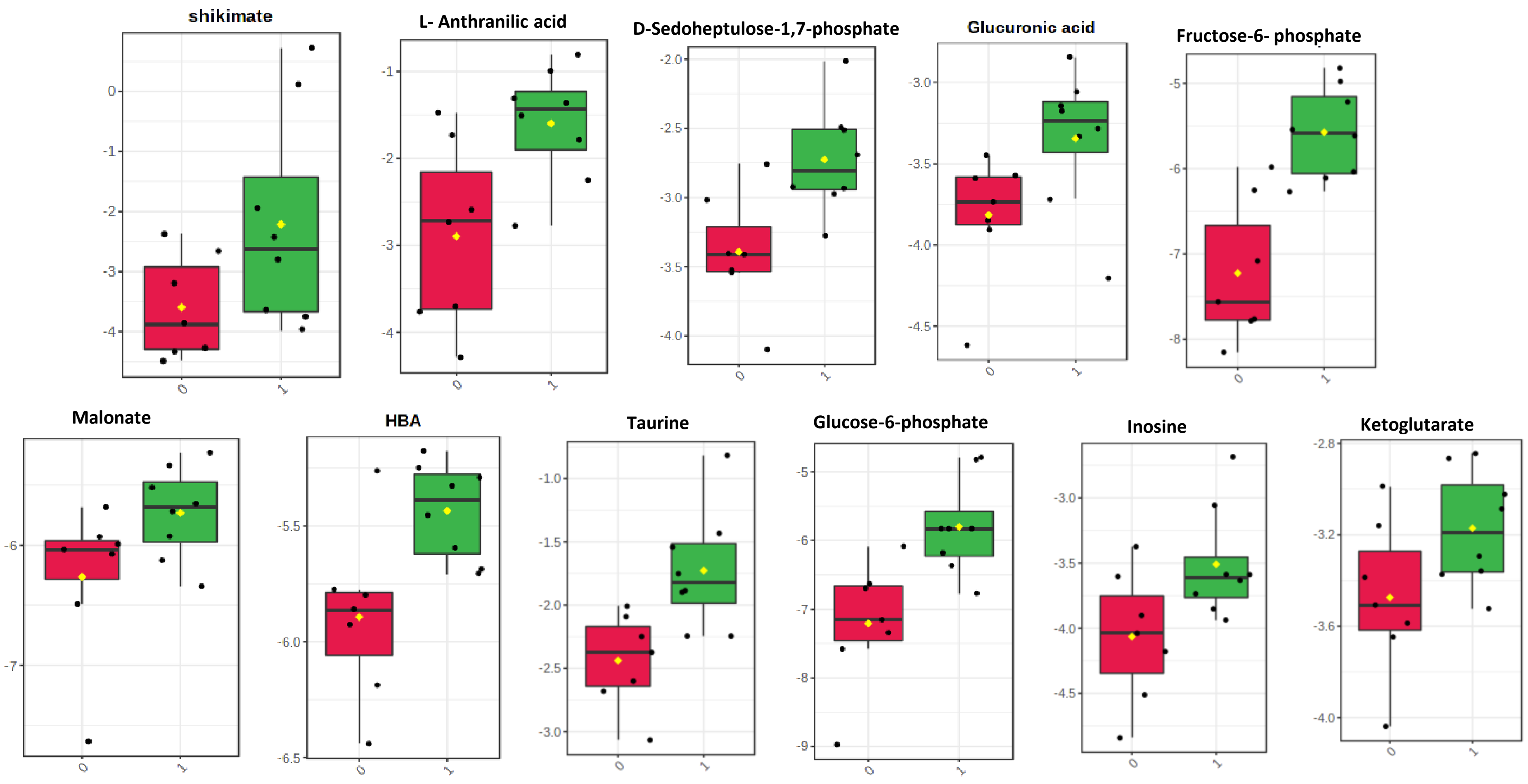

Supplementary Figure 3: showing 11 significant metabolites in the aqueous humor of PACG Patients compared to cataract controls

0- control

1- PACG

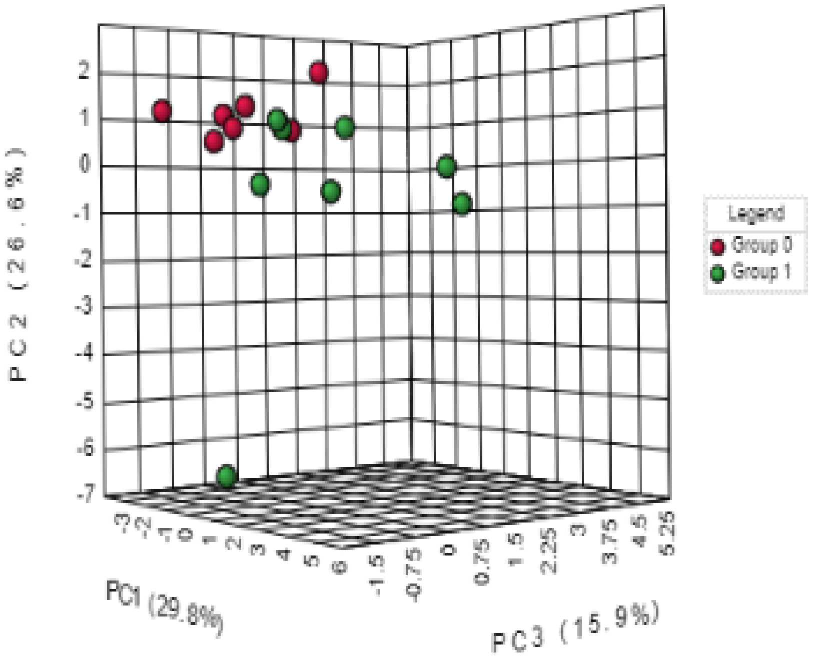

Supplementary Figure 3: PCA plot of the Control (red color) and PACG cohort (Green color)

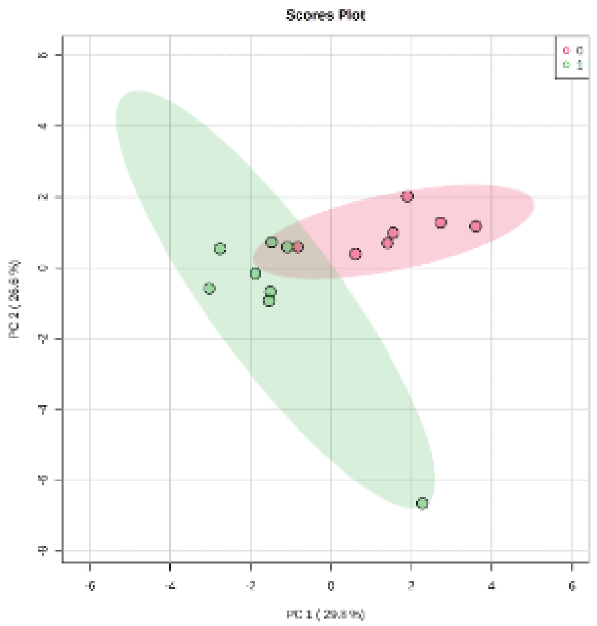

Supplementary Figure 3: showing score plot of the control and PACG cohort

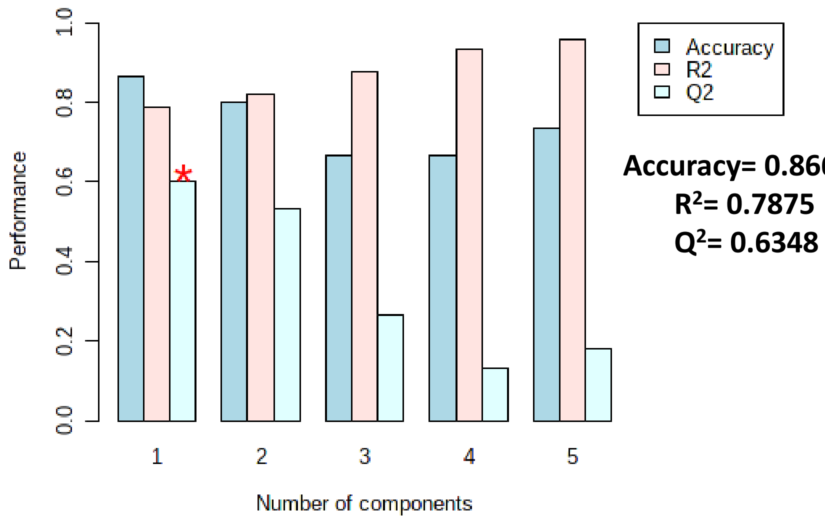

Accuracy= 0.8666
R^2= 0.7875
Q^2= 0.6348

Supplementary Figure 3: PLS-DA clustering anlaysis

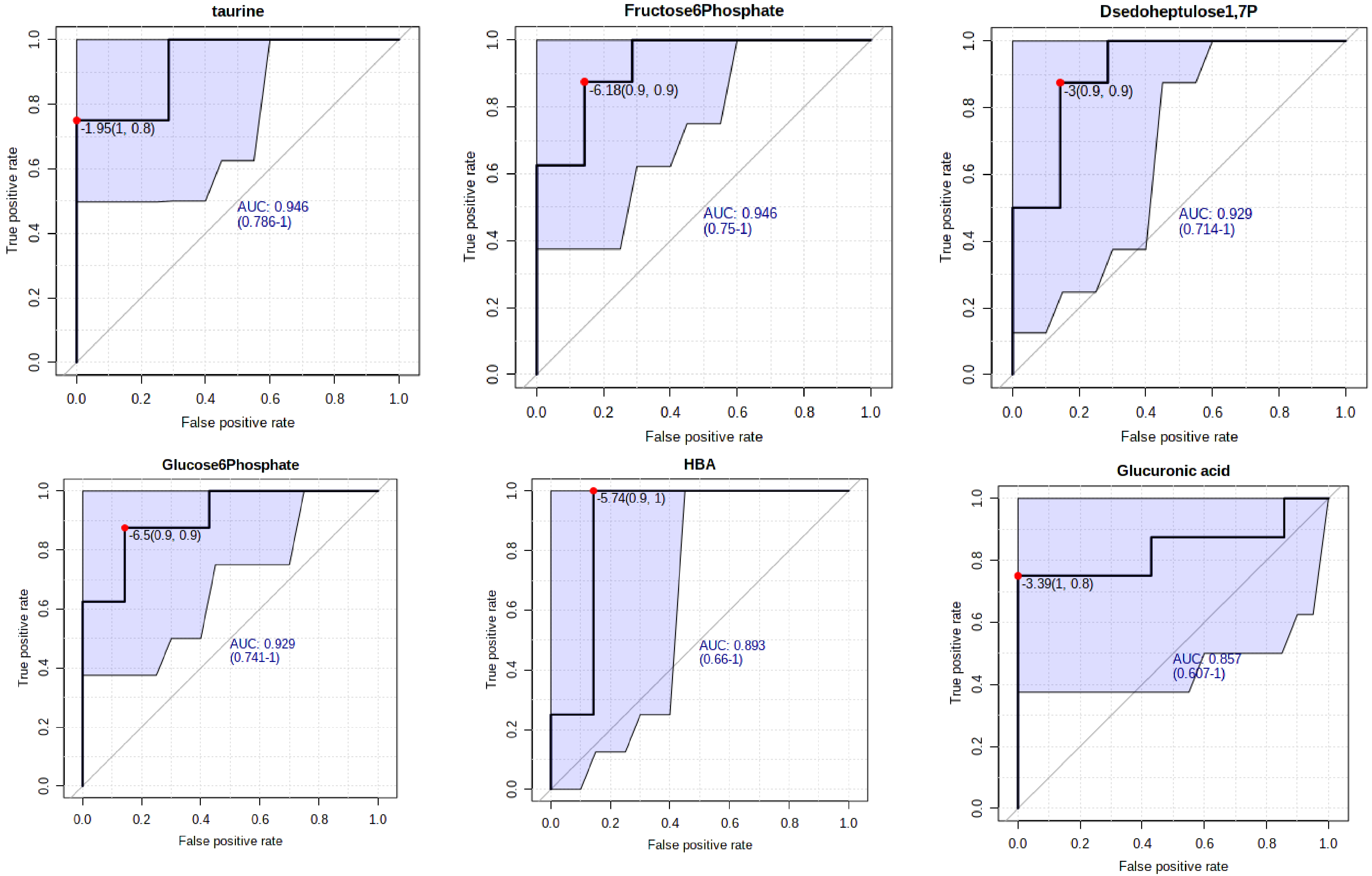

Supplementary Figure 3: Biomarker analysis in PACG patients.
