# Supplementary material for: Integrated clinical and metabolomic analysis identifies molecular signatures, biomarkers, and therapeutic targets in primary angle closure glaucoma
Source: Front Mol Biosci. 2024 Aug 9;11:1421030. doi: 10.3389/fmolb.2024.1421030 (PMC11341363; doi:10.3389/fmolb.2024.1421030)
Supplement: Supplementary file 7 [file Table3.pdf]

S.No	Metabolite	Reference
1.	Glucuronic acid	Studies have shown that glucuronic acid activates TLR4 and invoke ROS. (**Lewis et al., 2013).**
2.	Inosine	inosine and taurine are neuroprotective with implications for disease **(Hou B et al., 2004).**
3.	Taurine	inosine and taurine are neuroprotective with implications for disease (**Nor Arfuzir N et al., 2018)**
4.	Malonate	Malonate which is elevated in our PACG patient cohort was previously shown to induce cell death by collapsing the mitochondrial potential, invoking ROS (**Fernandez-Gomez FJ et al., 2005).**
5.	Hydroxybutyrate	Elevated levels of Hydroxybutyrate was found in the aqueous humor of PACG. Studies in mouse model of normal tension glaucoma have shown that every alternative day fasting leads to elevated levels of beta-hydroxybutyrate. Increased HBA lead to increased histone acetylation and upregulation of neurotrophic factors and catalase in the retina **(Brown JCC et al., 1986).**
6.	Alpha-ketoglutarate	Alpha-ketoglutarate which is elevated in the aqueous humor of PACG was found to be involved in the polarization of macrophage into the M2 phenotype and exert an anti-inflammatory response. **(Liu PS et al., 2017).**

Reference

1. Lewis SS, Hutchinson MR, Zhang Y, Hund DK, Maier SF, Rice KC, et al. Glucuronic acid and the ethanol metabolite ethyl-glucuronide cause toll-like receptor 4 activation and enhanced pain. Brain Behav Immun. 2013 May;30:24–32.
2. Fernandez-Gomez FJ, Galindo MF, Gómez-Lázaro M, Yuste VJ, Comella JX, Aguirre N, et al. Malonate induces cell death via mitochondrial potential collapse and delayed swelling through an ROS-dependent pathway. Br J Pharmacol. 2005;144(4):528–37.
3. Brown JCC, Sadler PJ, Spalton DJ, Juul SM, Macleod AF, Sönksen PH. Analysis of human aqueous humour by high resolution 1H NMR spectroscopy. Exp Eye Res. 1986;42(4):357–62.
4. Hou B, You SW, Wu MM, Kuang F, Liu HL, Jiao XY, et al. Neuroprotective Effect of Inosine on Axotomized Retinal Ganglion Cells in Adult Rats. Investig Ophthalmol Vis Sci. 2004;45(2):662–7.
5. Nor Arfuzir N, Agarwal R, Iezhitsa I, Agarwal P, Sidek S, Ismail N. Taurine protects against retinal and optic nerve damage induced by endothelin-1 in rats via antioxidant effects. Neural Regen Res. 2018;13(11):2014–21.
6. Liu PS, Wang H, Li X, Chao T, Teav T, Christen S, et al. A-Ketoglutarate Orchestrates Macrophage Activation Through Metabolic and Epigenetic Reprogramming. Nat Immunol. 2017;18(9):985–94.
