# Supplementary material for: Integrated clinical and metabolomic analysis identifies molecular signatures, biomarkers, and therapeutic targets in primary angle closure glaucoma
Source: Front Mol Biosci. 2024 Aug 9;11:1421030. doi: 10.3389/fmolb.2024.1421030 (PMC11341363; doi:10.3389/fmolb.2024.1421030)
Supplement: Supplementary file 8 [file Image1.pdf]

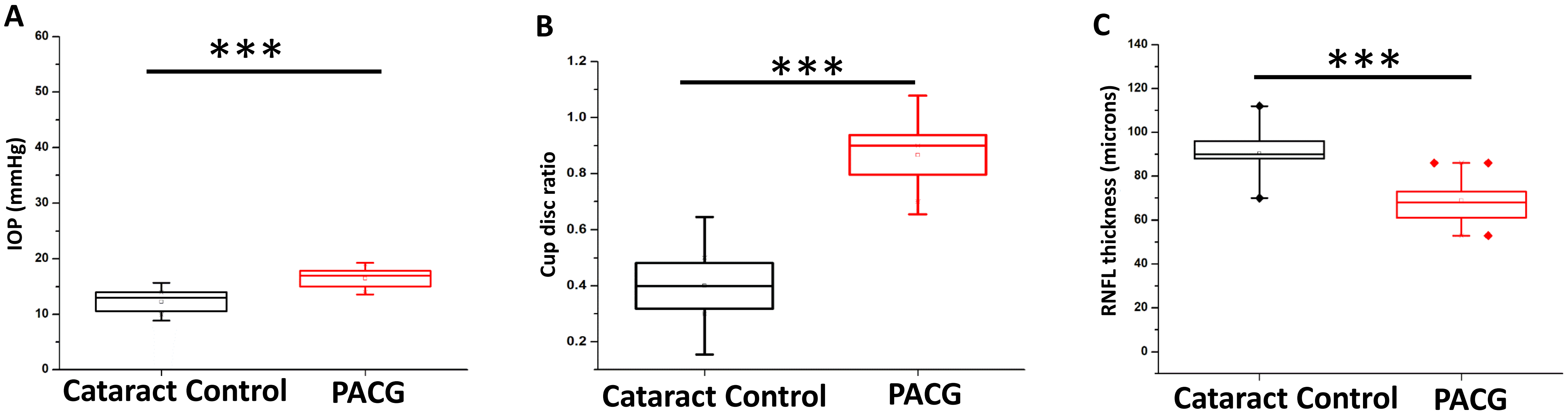

Supplementary Figure 1: Showing assessment of prospective clinical parameters **A.** The IOP profile in PACG group (n=9) compared to cataract control (n=7). **B.** The cup disc ratio in PACG group (n=9) compared to cataract control (n=7). **C.** Retinal nerve fiber layer thickness (RNFL) thickness in PACG patients (n=9) compared to cataract control eye (n=7).
